# Ordered release of genomic RNA during icosahedral virus disassembly

**DOI:** 10.1128/jvi.00053-26

**Published:** 2026-04-23

**Authors:** Yiyang Zhou, Andrew L. Routh

**Affiliations:** 1 Department of Pediatrics, Emory University 1371, Atlanta, Georgia, USA; 2 Department of Microbiology and Immunology, The University of Texas Medical Branch 547647, Galveston, Texas, USA; 3 Department of Biochemistry and Molecular Biology, The University of Texas Medical Branch 198643, Galveston, Texas, USA; 4 Department of Translational Medicine, Scripps Research 579993 , La Jolla, California, USA; 5 Sealy Center for Structural Biology and Molecular Biophysics, The University of Texas Medical Branch 430833, Galveston, Texas, USA; 6 Institute for Human Infections and Immunity, University of Texas Medical Branch 551582, Galveston, Texas, USA; Loyola University Chicago - Health Sciences Campus, Maywood, Illinois, USA

**Keywords:** icosahedral virus, RNA release, virus disassembly, PT-ClickSeq, RNA-capsid interaction

## Abstract

Many icosahedral viruses release their genomes through a series of ordered conformational changes involving distinct disassembly intermediates. While previous studies have focused on rearrangements of the capsid protein shell, it remains unclear whether viral genomes undergo defined structural transitions or are released in a specific order. Here, we developed “*PT-ClickSeq,*” a next-generation sequencing platform that natively profiles viral nucleic acids exposed during particle disassembly without RNA extraction or fragmentation. Using Flock House virus (FHV) as a model, which forms two well-defined disassembly intermediates, we found that FHV releases its RNA genome in a conserved, ordered manner: the 5′ and 3′ termini and specific internal loci are exposed first. Distinct genomic regions exhibited different energy barriers to release, suggesting a programmed exposure process. Complementary “vPAR-CL” analysis revealed progressive loss of RNA-capsid interactions, with RNA-protein interactions anti-correlated with genome release. Together, these findings show that the encapsidated genome actively orchestrates viral disassembly rather than serving as a passive cargo.

**IMPORTANCE:**

Viruses need to strike a balance between structural rigidity and flexibility to achieve both sufficient protection and rapid release of packaged genome into host cells. During the process of genome delivery, many viruses undergo a programmed disassembly process through successive morphological changes, which give rise to partially disassembled virus particles, termed disassembly intermediates. It is important to study these intermediates as “checkpoints” to understand virus disassembly dynamics. We established a next-generation sequencing method that can monitor the RNA behavior during these conformational changes. We found that different regions of RNA were released with different energy thresholds, and the RNA release prioritized regions with low RNA-protein interactions. These findings shed light on the active role of the viral RNA in virus disassembly.

## INTRODUCTION

Non-enveloped icosahedral viruses face a dual challenge: their capsid must protect the genome during transmission yet enable rapid release into the host cell at the right time. Switching between these two distinct roles is critical to avert premature exposure of the genomic cargo and to ensure its delivery to the correct cellular compartment (1). To accomplish this, viruses employ diverse mechanisms that trigger virus particle disassembly and genome release, such as receptor binding (2–5), compartmental pH changes (6–9), and other mechanical cues (1, 10, 11).

During the disassembly process, some viruses may proceed via an “*en masse*” approach, characterized by a heterogeneous and rapid disintegration of the capsid shell into component subunits (12, 13). Conversely, viruses may disassemble via a coordinated step-wise process, comprising successive conformational changes through multiple intermediate particles. This is demonstrated by polioviruses, rhinoviruses, and other enteroviruses, where transition from a full 160S virion to a 135S particle (or “A particle”) is triggered by receptor binding at physiological temperature during the early infection cycle. The “A particle” is further deconstructed into an 80S particle after RNA genome release (2). Icosahedral DNA viruses, such as adenoviruses, also can undergo a well-defined step-wise uncoating process during cellular entry (1, 14, 15). Similarly, multi-layered viruses, such as reoviruses and rotaviruses, sequentially lose capsid layers after receptor binding or during membrane penetration (16, 17). Single-layered icosahedral viruses may also shed specific capsid subunits (such as monomers, pentamers, hexamers, or others) to give rise to specific structural intermediates that feature a rupture or pore in the capsid shell through which the genomic cargo may egress (18, 19).

Disassembly intermediates provide insightful timestamps to characterize the spatiotemporal disassembly process of non-enveloped viruses. Prior studies have focused on the capsid proteins and their higher-order structures during disassembly. However, little is known about whether the genomic material is released in an ordered or structurally conserved manner. The transient and heterogeneous nature of intermediate particles presents a significant challenge in structural approaches, such as crystallography or cryoEM. Although high-resolution asymmetrical reconstructions can be achieved for disassembly intermediates, they often do not inform whether different genomic regions might be preferentially ordered and/or released during particle disassembly, nor the exact sequence identity underpinning observed RNA conformations.

Flock House virus (FHV, *Nodaviridae*) provides a model icosahedral virus for understanding non-enveloped virus assembly, disassembly, and RNA-capsid dynamics (20–23) and has well-documented disassembly intermediates in controlled experimental settings (24). The *T* = 3 icosahedral FHV particle stoichiometrically packages one molecule of each of the two genomic segments (RNA1 of 3.1 kb and RNA2 of 1.4 kb), which collectively encode only three viral proteins: (RNA-dependent RNA polymerase and protein B2 on RNA1, capsid on RNA2). Despite the simple particle structure and small genome, FHV virions contain extensive RNA-capsid interactions (23). During cell entry, the specific cell surface receptor for FHV has not been definitely identified, although receptor-mediated endocytosis is the most supported route. Following virus internalization, the lower pH of endosomal compartments, together with the depletion of Ca^2+^ ions, serves as the trigger for capsid conformational rearrangements (9, 25). These transitions result in the externalization of γ-peptide, which is a membrane-lytic peptide essential for disrupting the endosomal membrane and subsequent RNA genome translocation into the cytosol.

A recent study induced FHV particle conformational rearrangements with incremental heating *in vitro* and characterized with cryoEM the transformation from intact virions into “eluted” and a further “puff” disassembly intermediates (24). Interestingly, FHV “puff” intermediate particles showed further disintegration of the capsid layer and a partial RNA genome release through a twofold axis (hence the name “puff”).

Here, we used FHV as a model system to study the molecular mechanisms and events associated with RNA virus entry and particle disassembly. We developed a next-generation sequencing (NGS) technique termed Particle-Templated ClickSeq (“PT-ClickSeq”), which is based on the “ClickSeq” framework that avoids fragmentation or other pre-treatments of input RNA or DNA prior to NGS library synthesis. Therefore, PT-ClickSeq allows for NGS library preparation directly from unextracted biological materials such as virus particles and intermediates. Importantly, PT-ClickSeq preserves the viral RNA-capsid interactions in their native state, without risking artificial dissociation due to treatment such as RNase. With PT-ClickSeq, we found that the specific regions of the FHV genomic RNA are exposed in FHV disassembly intermediate particles. We demonstrated that the release of FHV RNA genome followed a step-wise pattern and that different genomic regions required distinct energy thresholds to be released. We further utilized viral photo-activatable ribonucleoside crosslinking (vPAR-CL) (23, 26) to characterize the RNA-capsid interactions during FHV disassembly. We found that the preferentially exposed loci lack significant RNA-capsid interactions. This indicates an active role for the viral genome in directing the choreographed structural dynamics of virus particle disassembly.

## RESULTS

### FHV disassembly intermediate particles are generated *in vitro* via heat treatment

**Fig 1:**
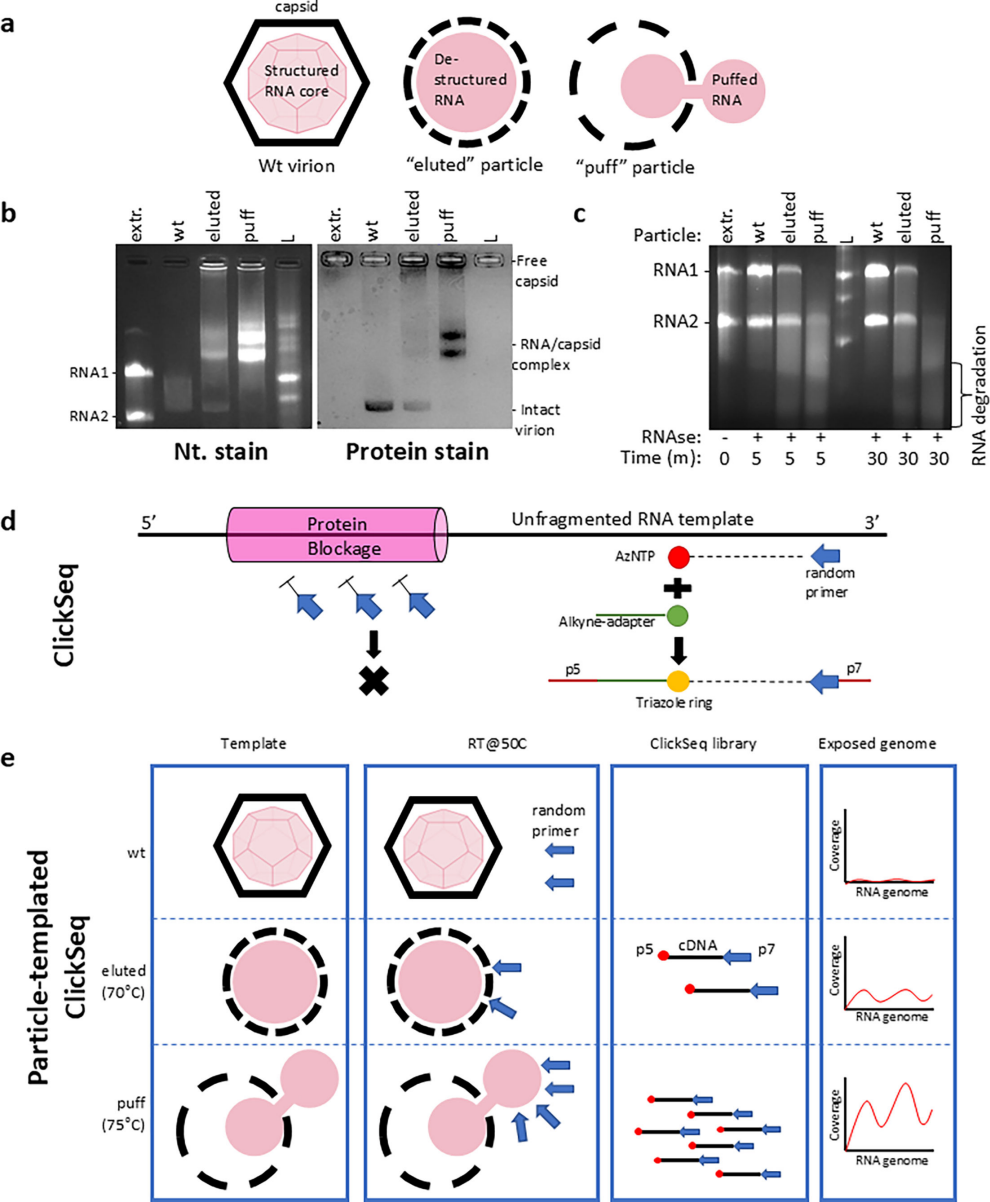
Sequencing of RNA exposed in disassembly intermediates of virus particles. (**a**) Schematic of FHV wt, “*eluted”* and “*puff”* particles. (**b**) Different particles show different permissiveness to nucleic acid staining in a non-denaturing RNA gel. Both eluted and puff particles form multiple RNA-capsid complexes, visualized using both nucleic acid stain (left) and protein stain (right). (**c**) The RNA genomes of different particles showed different susceptibilities to RNase A digestion. “Extr” = purified RNA extracted from wt particles. “L” = ssRNA ladder. (**d**) Schematic of the ClickSeq approach to assay exposed RNA. ClickSeq can sequence RNA without fragmentation. Therefore, ClickSeq can be adapted to sequence nucleic acids *in situ* to differentiate exposed RNA from protein-bound RNA or encapsidated RNA. (**e**) PT-ClickSeq uses RNA-protein complexes (such as an FHV puff particle) directly as the template during RT reaction to probe and sequence the exposed RNA regions. The different levels of RNA genome exposure are reflected by disproportionate sequence coverages (schematic presentation). “p5” and “p7” = Illumina sequencing adapter(s).

The formation of specific FHV disassembly intermediate particles can be induced upon controlled heating of wild-type virions *in vitro*, with two successive conformational transitions at 70°C and 75°C. These distinct structural states have previously been characterized by negative-stain transmission electron microscopy and cryo-electron microscopy with three-dimensional image reconstruction (24). Schematically (Fig. 1a), wild-type (wt) FHV comprises an ultra-stable, non-enveloped virion approximately 34 nm in diameter with extensive RNA-capsid interactions between the well-defined *T* = 3 icosahedral capsid shell and dodecahedral cage of encapsidated RNA (20, 23, 27, 28). Heating to 70°C yields particles with reduced density and dimension, forming “eluted” particles (24). When FHV virions are heated to 75°C, the capsid shell continues to lose structural integrity, and a portion of the encapsidated RNA genome can be observed extruding from the capsid shell to form “puff” particles (24).

In this study, we reproduce the generation of eluted and puff intermediate particles of FHV with controlled incremental heating, as described previously (24). Fifty micrograms of wt, eluted, and puff particles were directly loaded onto a 1% non-denaturing agarose gel pre-stained with nucleic acid dye (Fig. 1b, left). Intact wt virions exhibited little permeability to nucleic acid stain and appeared as a faint smear. This is consistent with previous thermostability studies of FHV (22). In contrast, eluted particles consisted of a mixture of intact wt virions and heterogeneous RNA-capsid complexes, which migrated slower than extracted and purified genomic RNA. The heterogeneity of the eluted particles is consistent with prior negative-stained low-resolution micrographs (24). In our assay, puff particles showed evidence of a continued disintegration from eluted particles. This is reflected by the increased nucleic acid stain of the RNA-capsid complex(es), altered migration pattern, and the absence of wt virions. Next, the same gel was post-stained with Coomassie Blue to evaluate the protein content of each sample (Fig. 1b, right). Puff particles exhibited stronger Coomassie staining than the eluted particles, likely due to the increased disorganization of capsids at higher temperatures. It is striking that, in spite of excessive heating (70°C/75°C for 30 min, respectively), eluted/puff particles retained the association of capsid protein with the viral RNA.

### FHV disassembly intermediate particles are structurally stable

In wt FHV, the intact icosahedral capsid shell protects the encapsidated RNA from harsh environmental conditions and antiviral host factors, such as naturally occurring RNases that would lead to genomic RNA digestion or fragmentation. We therefore sought to determine whether eluted or puff particles could retain resistance to RNase treatment. Following incremental heating and induction of eluted and puff particle formation, 50 µg of purified eluted or puff particles were treated with 5 µg of RNase A for 5 min at 37°C, and any remaining viral RNA was extracted thereafter and analyzed by electrophoresis on a non-denaturing agarose gel (Fig. 1c). In spite of RNase A treatment, eluted particles retained undigested, full-length RNA1 and RNA2, albeit at yields below that of wt virions, suggesting a partial protection of genomic RNA amongst the population of viral particles. In addition, eluted particles also showed a substantially greater degree of RNA degradation with evidence of nucleic acid products of lower molecular weight. This is consistent with the above-stated observation that eluted particles are structurally heterogeneous as a population. In contrast, puff particles retained only a small amount of full-length RNA2 after RNase digestion, with a higher intensity of lower molecular weight RNA smears relative to that of the eluted particles. This indicates that in puff particles, RNA1 is more susceptible to RNase digestion than RNA2, which suggests that RNA1 may be either less protected due to partial particle disassembly or due to a greater degree of externalization relative to RNA2 in puff particles. We further extended the RNase digestion to 30 min, which yielded similar trends to 5 min digestion. Importantly, this suggests that eluted and puff particles were relatively stable during this extended treatment and that digestion had reached its maximal completion, consistent with the notion that only specific portions of the viral genome are susceptible to RNase digestion in eluted and puff particles. These results demonstrate that FHV disassembly intermediates are structurally stable and exhibit altered conformational states of the packaged and exposed genomic RNAs.

### Particle-templated ClickSeq sequences exposed RNA in intermediate particles

PT-ClickSeq builds upon the ClickSeq platform for next-generation sequencing library synthesis that uses click-chemistry in place of enzymatic ligation for the addition of sequencing adapters (29, 30). In ClickSeq, a randomly-primed RT reaction is supplemented with 3′-azido-dNTPs that randomly terminate cDNA synthesis and release 3′-azido blocked cDNA fragments. Such 3′-azido-cDNAs are then “click-ligated” to a 5′-alkyne-functionalized sequencing adapter via click chemistry (copper-catalyzed azide-alkyne cycloaddition, CuAAC). After final PCR of the “click-ligated” cDNA, this process generates an NGS library with the required (e.g., Illumina) sequencing adapters and indexes (Fig. 1d). Importantly, unlike conventional NGS library preparation approaches, ClickSeq does not require nucleic acid fragmentation or similar pre-treatment of the input RNA template. Furthermore, the removal of other biological materials, such as cellular debris and viral or host proteins, prior to reverse transcription is also not required, so long as the nucleic acids are available for reverse transcription. Consequently, ClickSeq can accept a partially disassembled virus particle as input into the NGS library prep (Fig. 1e). This averts the denaturation of RNA-protein complexes during RNA isolation and retains the particle structure of disassembly intermediates. During RT, the exposed RNAs in disassembly intermediates allow the annealing of random hexamer primers, while the protected RNAs impede primer annealing. As a result, only exposed, unprotected RNAs can be successfully converted into a cDNA library and sequenced (Fig. 1d). The uneven genomic coverage of PT-ClickSeq thus reveals which regions of the genomic RNA are exposed in different disassembly intermediates of FHV.

As PT-ClickSeq generates signals only from exposed RNA, this “gain-of-signal” strategy also offers greater specificity than “loss-of-signal” approaches (e.g., RNase digestion of exposed RNA, followed by sequencing of the protected fragments). In a heterologous quasi-species, enzymatic reactions are often incomplete, leaving undigested templates that diminish the sensitivity and interpretability of “loss-of-signal” approaches.

### PT-ClickSeq reveals differential release of genomic RNA in disassembly intermediates

**Fig 2:**
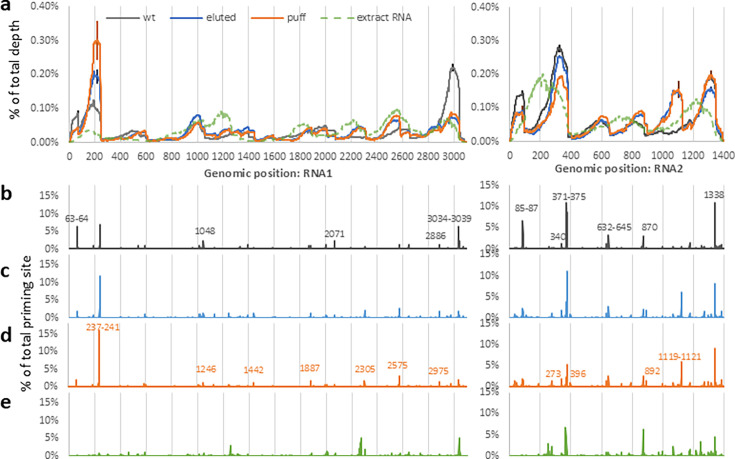
PT-ClickSeq reveals different genome coverages for disassembly intermediates. (**a**) Wt, eluted, and puff particles show distinct unevenness of read coverage for both RNA1 and RNA2. In contrast, extracted and purified genomic RNA is characterized by even and smoother read coverage across the genomes. With paired-end sequencing, the percentage of priming sites (**b–e**) on RNA1 and RNA2 is also consistent with overall genomic coverage (error bars: standard deviation, *N* = 3 biological replicates, only displayed on representative positions for clarity).

We first established the baseline of PT-ClickSeq read coverage over unencumbered, protein-free RNA. For this, we extracted and purified packaged RNA from wt virions and subjected it to canonical ClickSeq. The mapped reads were ratiometrically normalized to the total sequence depth. Consistent with prior NGS studies of FHV genomic RNA (31), the extracted FHV RNA demonstrated even read depth mapping across the viral genome (Fig. 2a, green dashed line), with expected minor undulations in read coverage indicative of biases inherent to NGS approaches due to viral primary sequence (e.g., GC content), uneven primer annealing, and/or RNA secondary structures.

After establishing this baseline, we sought to determine which regions of the FHV genomic RNAs were preferentially exposed in FHV disassembly intermediates. FHV wt (no-preheating), eluted (preheated at 70°C), and puff particles (preheated at 75°C) were directly used as input without RNA extraction in PT-ClickSeq (*n* = 3 for each condition). Libraries were successfully generated for each sample, although a lower yield of final NGS library for the FHV wt sample likely reflected the reduced availability of viral genomic RNA.

In contrast to the extracted FHV RNAs, sequencing of the viral disassembly intermediate samples revealed a stark unevenness in the distribution of sequence reads across the viral genomic RNAs, indicative of different genome exposure among wt, eluted, and puff particles for both RNA1 and RNA2 (Fig. 2a). For RNA1, the sequence read coverage for the wt particles showed enrichment at both 5′- and 3′-terminal regions. More specifically, RNA1: 26–75 nts (1.6% of RNA1 length) collectively accounted for 3.6% of total RNA1 coverage; RNA1: 151–250 nts (3.2% of RNA1 length) collectively accounted for 9.7% of total RNA1 coverage; RNA1: 2,901–3,050 nts (4.8% of RNA1 length) collectively accounted for 23.6% of total RNA1 coverage (Fig. S1a and Table S1). In contrast, the read coverage of extracted and purified genomic RNA was relatively smooth and showed less enrichment in comparison to virus particles (wt, eluted, or puff) (Fig. S1a and Table S1). As the coverage information was normalized to the percentage of total sequenced depth, this suggests that the 5′ and 3′ termini of the viral genome were disproportionately exposed in wt particles compared to the rest of the genome.

Similarly to wt FHV, both eluted and puff particles showed increased coverage at the 5′ terminal 26–75 nts (1.8% of total coverage) and 3′-terminal 2,901–3,050 nts (9.0%–9.7% of total coverage) of RNA1 relative to the extracted FHV RNA (Fig. S1a and Table S1). The most dramatic increase in read coverage was observed at 151–250 nts of RNA1, accounting for 16.8% and 23.3% of the total mapped reads for eluted and puff particles, respectively, while only 2.7% of the reads mapped in this region for the extracted FHV RNA (Fig. S1a and Table S1). Comparing wt particles with eluted/puff particles, we also detected increases in read coverage at 2,200–2,300 nts and 2,425–2,725 nts in both eluted and puff particles.

In RNA2, wt FHV particles again showed disproportionally high coverage at 5′ terminal 26–100 nts (5.4% of RNA2 length and 8.9% of total RNA2 coverage), 276–375 nts (7.1% of RNA2 length and 24.9% of total RNA2 coverage), and the 3′ terminus 1,251–1,350 nts (7.1% of RNA2 length and 16.2% of total RNA2 coverage) (Fig. S1a and Table S1). In contrast, eluted and puff particles exhibited a substantial increase in coverage in RNA2: 1,051–1,150 nts (wt: 3.6%, eluted: 11.8%, puff: 11.7%), and a slight increase in 800–875 nts.

Importantly, the disproportionate enrichment of sequence data over these regions is not seen, or read coverage is flattened in the extracted FHV RNA samples over the same regions. This provides confidence that the observed enrichment is due to the increased exposure of released RNA and de-encapsidation of RNA in the disassembly intermediates, rather than due to artifacts and biases of NGS library prep and PCR. To further demonstrate the above-stated observations, a moving average map (50 nt window) of genomic coverage and the collective coverage of specific genomic regions is provided in Fig. S1b.

### PT-ClickSeq identifies priming sites of exposed RNA

We further investigated whether the increased genome coverage (Fig. 2a) is a result of RNA exposure and increased priming in puff particles. Since the PT-ClickSeq data were paired-end, the 5′-most coordinates of the “R2” read of the paired-end data correspond to the priming sites during the reverse transcription reaction (Fig. S2, priming site is defined here as the nucleotide position immediate upstream of the 3′ random hexamer sequence). The normalized priming site depths (Fig. 2b through e) exhibited trends consistent with the total read coverage data (Fig. 2a), as expected. Several abundant priming sites were located directly downstream of regions exhibiting high genomic coverage. These paired-end data further demonstrate that the coverage differences in wt, eluted, and puff particles were led by the different RNA availability for primer annealing.

The read coverage and priming site data obtained by PT-ClickSeq provide several insights into FHV disassembly intermediates and genome release. First, the differential exposure of genomic cargo at low temperatures in wt particles (50°C during RT incubation) suggests that the FHV RNA genome 5′ and 3′ proximal regions may be more susceptible to ambient thermodynamic fluctuations and thus are prone to be released from the capsid shell at lower temperatures. This dynamism of viral particles, canonically considered to be completely rigid, is consistent with mass-spectrometry analyses that demonstrate the transient exposure of the “gamma” peptide of the capsid protein that is nominally located on the interior of the capsid shell to exogenous proteases even at ambient temperatures (32). Second, eluted and puff particles showed generally consistent and conserved profiles, suggesting that genomic RNA release may prioritize specific regions during disassembly in a programmatic manner rather than simply releasing the entire genome all at once “*en masse*” or in a non-conserved disorganized fashion. Third, compared to eluted particles, puff particles only showed a substantial increase in coverage at RNA1: 151–250 nts, suggesting that RNA1: 151–250 nts may be the component of the “puffs” observed in previous CryoEM studies (24).

### Different genomic regions must overcome specific energy barriers prior to release during virion disassembly

**Fig 3:**
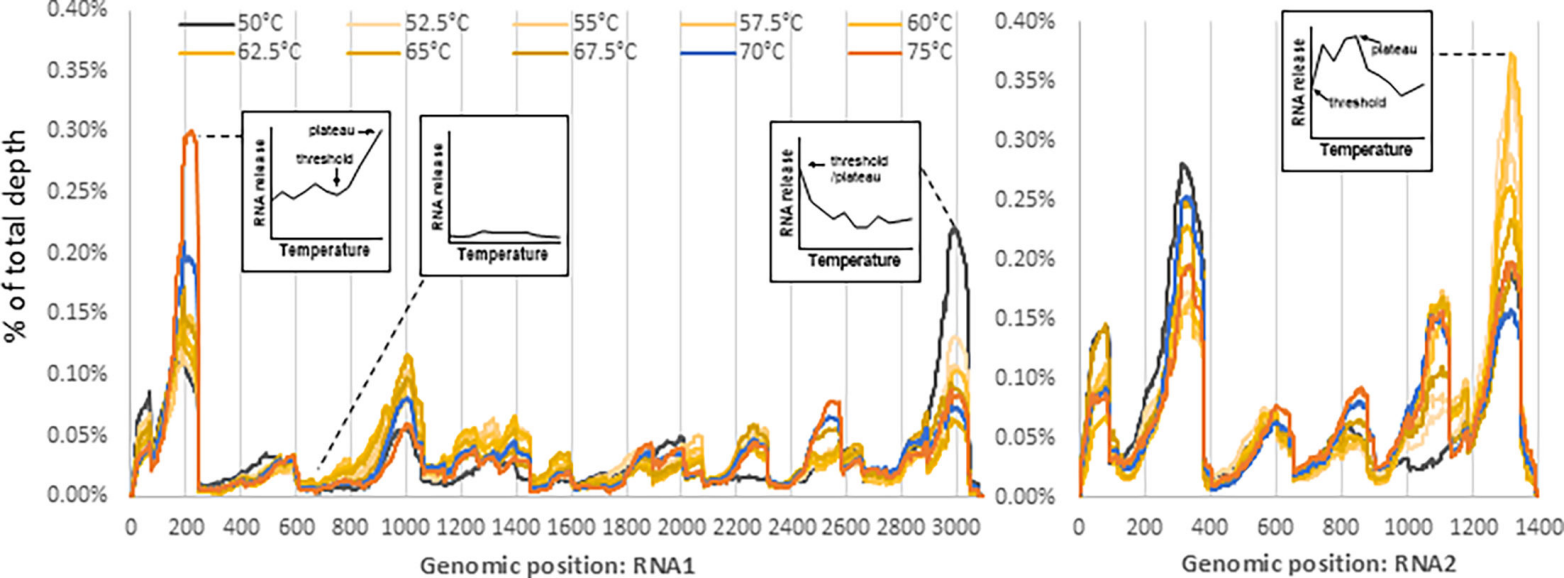
PT-ClickSeq reveals release of specific genomic regions at different temperatures. Different FHV genomic regions show differential exposure following heat treatment at temperatures ranging from 50°C to 75°C (50°C = wt particle without pre-heating, 70°C = eluted particle, 75°C = puff particle, normalized to the % of total depth).

To extend PT-ClickSeq beyond the eluted and puff particle intermediates and to characterize the dynamics of particle disassembly and genome release, we heated purified FHV virions with incremental increases in temperature treatment ranging from 52.5°C to 67.5°C, with 2.5°C intervals. After heat treatment, virus particles were placed on ice until further characterization. We then performed PT-ClickSeq and evaluated the read coverage over the viral genome to reveal genomic RNA exposure (Fig. 3). As before, read coverage was normalized to the total sequenced depth across the viral genome.

We observed that for both genomic RNAs, specific genomic loci showed distinctive trends in read coverage at different temperatures: (i) a sequentially increased coverage correlating with temperature (e.g., RNA1: 90–250 nts, RNA2: 1,000–1,130 nts), indicating continually increased exposure of RNA upon increased temperature treatment; (ii) generally negative correlation (e.g., RNA1: 1–75, RNA1: 2,900–3,040 nts), indicating maximal relative exposure early during disassembly and thus *proportionately* reduced read coverage upon treatment at higher temperatures; (iii) unchanged regions of low coverage (e.g., RNA1: 545–700 nts, RNA2: 400–570 nts), indicating consistently protected genomic loci; (iv) regions of fluctuating coverage during the range of the heat treatment (e.g., RNA1: 950–1,060 nts, RNA2: 1–90 nts), suggesting a mid-to-early exposure of viral RNA during an intermediate stage of disassembly. The representative regions, genomic positions, and profiles of the PT-ClickSeq data are presented in Fig. S3.

This information provides insights into the different energy barriers that must be overcome for each genomic region to be exposed. For example, RNA1: 2,150–2,310 exhibited a ~3.1-fold increase in coverage from 50°C to 52.5°C, which was followed by a fluctuation in coverage between 52.5°C and 75°C. This suggests an early release of this genomic region upon heat treatment. In contrast, RNA1: 2,500–2,580 nts exhibited minimal changes in read coverage upon heat treatment between 50°C and 65°C, but a subsequent increase in read coverage after 67.5°C. The coverage of RNA2: 1,250–1,350 nts was relatively unchanged when comparing wt, eluted, and puff particles (Fig. 2). However, upon further investigation, substantially increased genome release can be detected from 50°C to 52.5°C, which remained at a plateau from 52.5°C to 60°C and then gradually decreased thereafter (Fig. 3, Fig. S3). This indicates that the release of RNA2 3′-terminus overcomes the first energy barrier of 52.5°C and continues to release RNA until genome release peaks at 60°C. The decrease in coverage at temperature treatments >60°C is due to the continued relative increase of other genomic regions during virus particle disassembly.

These data exemplify how different genomic regions, even those separated by less than 200 nts of genetic distance, can exhibit different energy barriers prior to their exposure during virus particle disassembly. Altogether, these data support a model for the release of genomic RNA from FHV particles via an ordered and step-wise process, rather than a disordered or “*en masse*” action of release.

### RNA-protein interaction disorganization in FHV disassembly intermediates revealed by vPAR-CL

vPAR-CL is an NGS-based high-throughput method developed to study RNA-protein interactions in the context of intact virus particles (23, 33). Briefly, cells in culture are supplemented with 4-thiouridine (4SU), which is subsequently incorporated into viral genomic RNA during replication. Purified virions with RNA genome(s) containing 4SU can be UV crosslinked to adjacent protein side chains of the viral capsid shell. During RNA-seq library generation, the resultant RNA-amino acid adduct can be read through by the reverse transcriptase, but inserting a guanine opposite the crosslinked uracil rather than an adenine. As a result, U-C transitions can be enumerated in the final NGS data sets, revealing the exact nucleotide position of RNA-protein interactions. Unlike chemical crosslinkers (such as formaldehyde), 4SU/UV-induced crosslinking is limited to “zero distance” (does not add an atom) RNA-protein interactions (26), which places vPAR-CL as an ideal method to characterize the RNA-capsid interaction sites in mature virions or during the disassembly process. Importantly, vPAR-CL does not rely on the enrichment of crosslinked RNA-protein adduct (in contrast to similar approaches such as PAR-CLiP [34] and derivatives [26]). Instead, the U-C transition rate (the “vPAR-CL signal”) is a ratiometric measure of the presence and consistency across a population of conserved RNA-protein interactions. This also places vPAR-CL as a suitable approach to monitor genome-capsid dynamics. As a result, the expected loss of RNA-capsid interaction during the FHV disassembly process will be reflected by diminished U-C transition rate across the viral genome or at specific regions, rather than the loss of overall sequence coverage.

**Fig 4:**
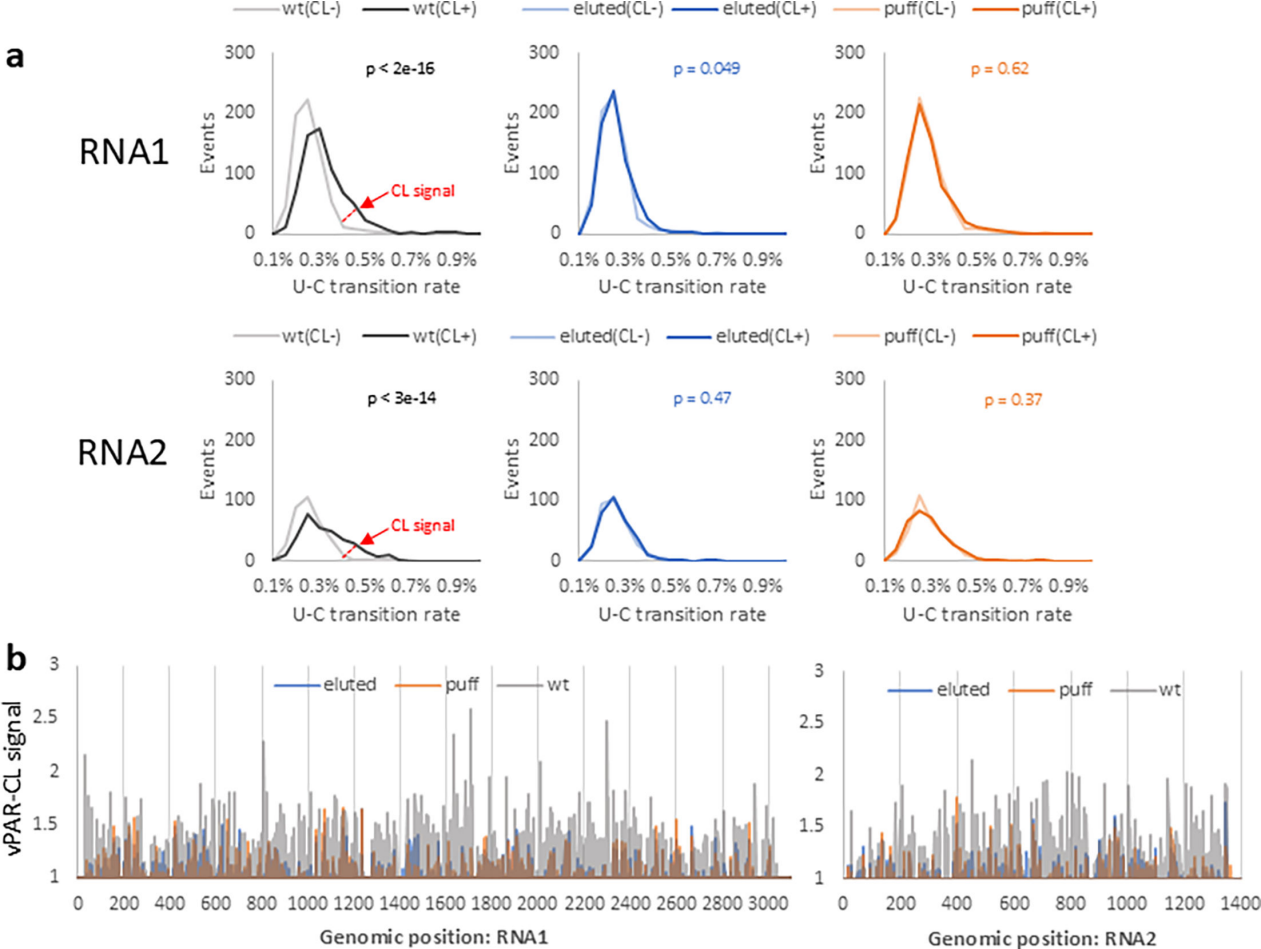
vPAR-CL signals of different disassembly intermediates reveal different RNA-capsid interaction landscapes. (**a**) Among different particles, the crosslinked wt particle [“wt(CL+)”] elicited a higher U-C transition rate than that of the control [“wt(CL−)”], which indicates robust crosslinking at RNA-capsid interaction sites. In contrast, the U-C transition rates of eluted or puff particles were comparable to their respective controls. The average U-C transition rates of all U positions were compared between CL+ and CL−, by two-sample Kolmogorov-Smirnov tests (*N* = 4 biological replicates). (**b**) The vPAR-CL signals of wt, eluted, and puff particles showed overlapping RNA-capsid interaction patterns between eluted and puff particles, but contrasting to the signals of wt particles. Signals were generated from the average of four independent experiments.

As an important validation of the approach, we first assayed the U-C transition rate in the same virus particle samples before (“CL−”) and after (“CL+”) UV crosslinking (Fig. 4a). As expected, wt particles demonstrate increased rates of U-C transitions in the CL+ samples than CL− controls (confirmed by two-sample Kolmogorov-Smirnov [K-S] tests, *P* < 2e-16 for RNA1 and *P* < 3e-14 for RNA2). We observed that wt FHV elicited strong and widespread vPAR-CL signals at multiple loci across both RNA1 and RNA2 (Fig. 4b, Fig. S4). In contrast, the eluted and puff particles both showed diminished overall U-C transition rates (K-S *P* values > 0.049).

Having established that crosslinking induced specific U-C transitions, we investigated the vPAR-CL signals (positional fold change of U-C transition rate of CL+/CL−) of different particles. Compared to the extensive vPAR-CL signal of wt particles, eluted and puff particles exhibited a clear reduction in vPAR-CL signals across both RNA1 and RNA2 (Fig. 4b, Fig. S4). This suggests loss of conserved RNA-capsid interactions in eluted and puff particles. Globally, the vPAR-CL signals of eluted and puff particles exhibited a strong linear correlation (Pearson r = 0.78) (Fig. S5). In contrast, there was little or no correlation of vPAR-CL signals between puff and wt particles (Pearson r = 0.0047). The overlapping vPAR-CL signals between eluted and puff particles are important, as this suggests that despite the previously observed morphological differences, the eluted and puff particles exhibit similar internal RNA organization (or lack thereof), and the resultant internal RNA-capsid interactions were consistently retained through morphological transformation and RNA genome release.

Altogether, these data indicate significant re-ordering of encapsidated RNAs and dramatic loss of RNA-capsid interactions in these particles. It is important to note that, although eluted and puff particles exhibited generally reduced vPAR-CL signals compared to wt, several genomic positions retained comparable or even increased vPAR-CL signals (e.g., RNA1:U1233, RNA2:U400). This is consistent with the electrophoretic mobility shift assay (Fig. 1b) and suggests that in spite of extensive heating, some virion RNA-capsid interactions are retained.

### FHV genome release during disassembly is anti-correlated with RNA-capsid interactions

**Fig 5:**
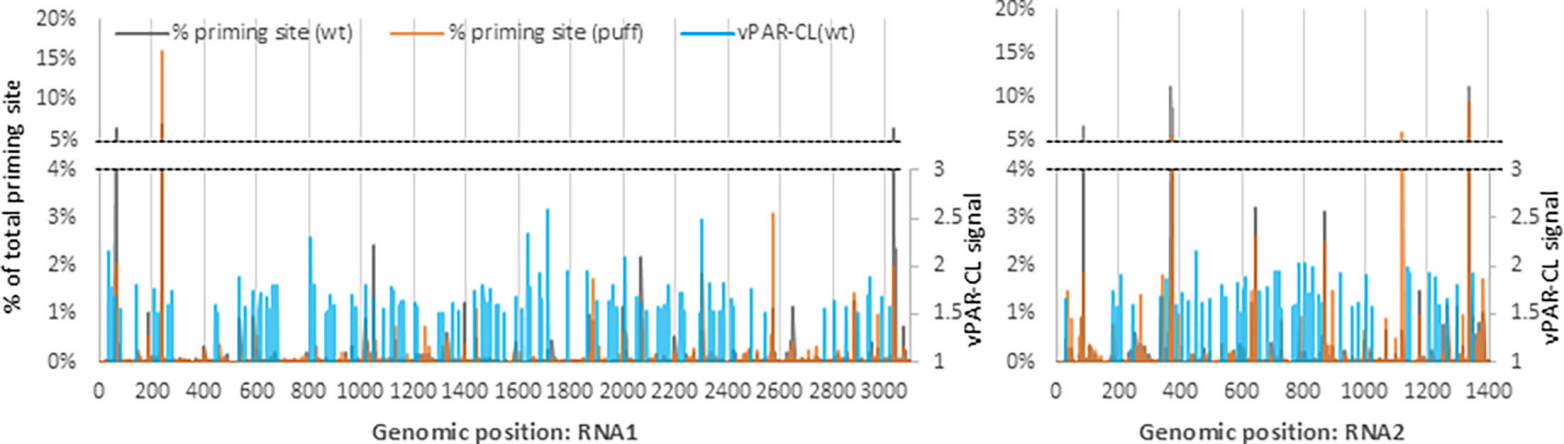
Anti-correlation between exposed genomic regions and RNA-capsid interactions. The exposed genomic regions in wt and puff particles are indicated by the % of total priming sites. These exposed sites are often characterized by a lack of U’s in the sequences immediately downstream, or a lack of meaningful RNA-capsid interactions (vPAR-CL signal <1.5).

The formation of FHV disassembly intermediates demonstrates strong RNA-capsid interactions that survived extensive heating (Fig. 1). We sought to understand whether the RNA-capsid interactions in wt FHV contributed to the differential RNA genome release during the virus disassembly process (Fig. 2). We compared the vPAR-CL signals of wt particles (>1.5; correspondent to the top 15% vPAR-CL signals in wt FHV, Fig. S4) to the priming site data of wt and puff particles from PT-ClickSeq (Fig. 5). We found that preferentially released RNA loci were characterized by a substantial reduction in vPAR-CL signals. This is demonstrated in Table S2, which reports the PT-ClickSeq priming sites and vPAR-CL signals in wt and puff particles for representative sites in RNA1 and RNA2. We also investigated the correlation between priming rates and vPAR-CL signals. A moving average (15 nts) priming percentage and vPAR-CL signals in wt particle showed a moderate but significant negative Spearman correlation coefficient (r = −0.35, ANOVA *P* = 1E-90 for RNA1, r = −0.27, ANOVA *P* = 6E-24 for RNA2), suggesting that the RNA genome release at lower temperatures (50°C) favors sites without conserved RNA-capsid interactions. Similarly, the differentially exposed sites in puff particles also exhibited significant anti-correlation with vPAR-CL signals (moving average with 15 nts window, r = −0.27, ANOVA *P* = 5e-54 for RNA1 and r = −0.31, ANOVA *P* = 1e-31 for RNA2).

## DISCUSSION

In this study, we used FHV as a model to understand programmed viral RNA release. We used incremental heating to recreate FHV intermediate particles and characterized the molecular dynamics of FHV “elute” and “puff” particles. We describe the novel next-generation sequencing method “Particle-Templated ClickSeq” that can be used to sequence partially disassembled viral particles in the native state. We found that FHV genome release prioritizes the 5′ and 3′ termini and other select genomic regions. Different genomic sites also overcome different energy barriers prior to genome release. These prioritized sites correlate with regions that lack significant RNA-protein interactions identified via vPAR-CL.

High-resolution structural studies of intermediate particles of icosahedral virus assembly and disassembly present a significant challenge for advanced imaging technologies, such as CryoEM, due to their transient nature and conformational heterogeneity. Furthermore, it is often difficult to gain information regarding the encapsidated genetic cargo or the dynamic relationship between RNA and capsid during virus disassembly. Recent studies of virus particles and disassembly intermediates have revealed that the encapsidated viral genome is often asymmetrically ordered within the capsid shell in spite of the imposing structure of the icosahedral capsid shell (24). In this manuscript, we re-purposed a fragmentation-free RNA-seq method, “ClickSeq,” and demonstrated that it can be used as a convenient tool to study icosahedral virus disassembly intermediates and reveal the sequences of exposed RNA, as well as the energetic barriers to release for different genomic regions. “PT-ClickSeq” uses partially disassembled particles as input for NGS library synthesis. This allows probing of exposed RNA under native conditions. FHV disassembly intermediates are induced through heat treatment at 70°C/75°C. Importantly, these conditions are “harsher” than the reverse transcription conditions (extension occurs at 50°C), and the elute and puff particles are demonstrated to withstand the reverse transcription conditions. This allows us to adopt PT-ClickSeq in a simple manner without having to alter and optimize reverse transcription reaction conditions at lower temperatures. In contrast, RNA footprinting-based probing methods (e.g., RNase digestion of exposed RNA) may disrupt the structural integrity of FHV intermediates, altering the structural dynamics of RNA-protein relations during FHV disassembly.

During virus entry and subsequent disassembly, virus particles undergo numerous coordinated and concerted *in vivo* conformational changes that are triggered and dependent upon specific interactions between virus particles and intracellular host factors (e.g., receptor-induced viral uncoating, pH changes in the endosome, ionic conditions, translation-mediated genome unpacking by the host ribosomes). These interactions and factors alter the energy landscape of the virus particle in a choreographed manner that results in specific and ordered structural/morphological changes. In this study, FHV disassembly intermediates were obtained by heating wt virions to 70°C or 75°C. Although *in vitro* heat treatment of purified virus particles does not replicate intracellular physiological conditions, such heating of viruses provides a simple and well-controlled method to recapitulate the incremental structural changes that occur during virus particle disassembly under *in vitro* conditions. It has been demonstrated in poliovirus that the conformational changes during heat-induced uncoating fully recapitulate those *in vivo* after receptor binding (35). Heat-induced virion structural changes have also been studied among DNA phages (36–38). In a previous study, a heat-induced FHV RNA-RNA interaction was demonstrated to be important for virus packaging and virion integrity (22).

In this study, using PT-ClickSeq, we revealed that the heat-induced FHV RNA genome release follows a step-wise order and that different genomic regions must overcome different thermodynamic barriers to be exposed (Fig. 2 and 3). During the transition from intact virions to disassembly intermediates (“eluted” and “puff” particles), only specific regions of the RNA genome showed significantly increased accessibility. This observation contrasts with a possible “*en masse*” model wherein the genetic material is released altogether in a disordered manner. Such a model would result in different genomic loci being randomly and simultaneously exposed across the virus particles that make up the whole ensemble population, which would yield even read coverage across the viral genome without the features we observed in our study. Further evidence of FHV genome packaging and release can be demonstrated by the vPAR-CL experiments that showed that even under escalated heating conditions, the RNA-capsid interactions are not completely disordered. Instead, RNA-capsid interactions survived the disruptive heat, and consistent structural tropism was still retained in eluted and puff particles (Fig. 4, Fig. S5).

From our PT-ClickSeq experiment (Fig. 2 and 3), it is particularly interesting that the 5′ and 3′ termini for both RNA1 and RNA2 are disproportionately exposed at lower temperatures (50°C), where the virus particle is expected to remain fully intact. This observation is reminiscent of the transient exposure, or “breathing” of the membrane-penetrating “gamma-peptide” at ambient temperatures when observed by mass spec and in computational modeling approaches (32, 39). Similar observations have been demonstrated by a common cold virus (human rhinovirus 2), which is highlighted by the rapidly accessible 3′-end of viral RNA to hybridization methods during heating (40). These findings suggest a mechanism of icosahedral virus genome egress whereby the free termini of each RNA encounter lower energy barriers to release than the rest of the genome and therefore can be preferentially exposed during cell entry. It has been proposed that the ends of large ssRNA (such as that of a virus) are necessarily close (41). In this study, the concurrent 5′ and 3′ genome release also suggests that FHV RNA genome termini may establish spatially close interactions in virions.

Functionally, it is also conceivable that the rapid accessibility of viral RNA termini plays essential roles in virus replication after cell entry: the 5′-end of +ssRNA viral RNA mimics host mRNAs to recruit the host ribosomal subunits; the 3′-end of +ssRNA virus genome typically serves as an initial template for viral genomic or subgenomic RNA replication. Certain characteristics of viral +ssRNA 3′ end (such as polyadenylation) may also function to ensure viral genome stability or to prevent cellular exoribonuclease cleavage (42–44). For FHV, the 5′- and 3′-regions of the viral RNA also contain important functional motifs required early during the infection cycle: FHV RNA1 5′-region contains an important cis-acting element which directs RNA replication to the mitochondria membrane, while the RNA1 3′-end contains the subgenomic RNA3 that encodes silencing suppressor protein B2 (45, 46). The early released RNA1: 2,900–3,100 nts region identified in this study also coincides with FHV 3′ *cis* elements that are important for the replication of RNA3 and RNA2 (47, 48). In comparison to RNA1, the motifs of FHV RNA2 are less well characterized. However, the early released RNA2: 1–370 nts region contains a stem-loop structure that is important for RNA2 genome packaging and an RNA1-RNA2 heterodimer stem, which are shown to be important for genome packaging specificity, as well as overall virion thermostability (22). RNA2: 1,200–1,400 nts region also contains a 3′ *cis* element, responsible for regulating RNA2 replication (49).

In addition to energy thresholds and pH shifts that govern FHV disassembly, virion disassembly can also be impacted by ionic conditions at different stages of infection. Previous work with picornaviruses provides clear evidence that monovalent cations modulate energy landscapes of uncoating and genome release in a pH-dependent manner (50). Endosomal ions further act to stabilize uncoating intermediates by impacting RNA-protein contacts (51, 52). The endosomal conditions (e.g., electrostatic interactions, counterion binding, local ionic strength, and pH) collectively fine-tune the virion structural determinants to orchestrate genome uncoating (53). By analogy, we anticipate that FHV particles will also exhibit distinct uncoating programs under different ionic conditions that mimic early or late endosomes. Future systemic experiments that vary monovalent and divalent ion composition and pH will be essential for defining how chemical cues may synergize with energy changes to influence RNA-capsid dynamics and to regulate FHV disassembly and RNA release.

Other evidence also suggests that FHV genome release is a gradual, step-wise process instead of the abrupt disordering of virus particles. Conformational studies show that FHV genome release may be confined to a single portal at a single twofold axis (24), which reflects the presence of ordered RNA duplexes at the same axis in virus particles (20). In addition to FHV, the egress of genomic RNA from a single pore on a virus particle has also been demonstrated in other icosahedral viruses, such as flaviviruses (18), poliovirus and human enteroviruses (35, 40, 54–57), as well as simulated for virus-like particles (58). The highly symmetrical nature of non-enveloped virus particles (e.g., an icosahedral particle would encompass 15 identical twofold axes) begs the curious question of how the virus designates one portal/axis for genome release. We posit that the asymmetrically ordered sequence(s) and specific RNA-capsid interactions (or the lack thereof) within a virus particle dictate the order of genome release from the capsid shell. As such, the encapsidated RNA genome is not a passive cargo but rather plays an active and ordered role in directing the structural changes required for a virus to successfully infect a host cell (59).

## MATERIALS AND METHODS

### Cell culture and virus stocks

wt FHV was generated by co-transfecting *Drosophila melanogaster* (S2) cells with pMT plasmids containing either FHV RNA1 or RNA2. FHV gene expression was induced with copper sulfate 24 h after transfection. The transfected cells (P0) were used to inoculate naïve S2 cells to generate continuous passages (P1 or P2) of virus stocks. In this study, all FHV stocks used are either P1 or P2 viruses to preserve full-length genomes without accumulation of defective viral genomes (31). For purification of virus particles, wt FHV stocks were sequentially purified with 4% polyethylene glycol 8000 precipitation, RNase/DNase digestion, sucrose gradient ultracentrifugation, and 100 K MWCO polyether sulfone (PES) membrane filtration. The detailed methods regarding cell culture maintenance, virus passaging, transfection, and purification are as previously described (22, 23).

### FHV disassembly intermediates

The heat-induced FHV disassembly intermediates (namely “eluted” and “puff” particles) were generated using previously established methods (24). Briefly, 50 µg of purified wt FHV particles were resuspended in 50 mM HEPES (pH 7.4, Na^+^ as counter ion) and stored at 4°C. During heating, a thermocycler program was set to heat the wt virions from 4°C to the inducing temperatures (70°C for the eluted particle and 75°C for the puff particle) at a ramp rate of +0.1°C/s. The particles were then continuously heated at the inducing temperatures for 30 min and placed on ice immediately thereafter.

### Agarose gel electrophoresis

All electrophoresis experiments were conducted under non-denaturing conditions. Purified viral particles (wt, eluted, or puff) were directly loaded (Fig. 1b) without RNA extraction. Electrophoresis was conducted with 1× lithium acetate borate buffer and 1% non-denaturing agarose gel pre-stained with GelStar (Lonza) to visualize nucleic acid content first. To visualize protein content, the same gel was then post-stained with Coomassie Blue (R-250) and destained with standard Coomassie destain (40% methanol, 10% glacial acetic acid), as previously described (60). RNA (Fig. 1c) was extracted from virus particles after RNase digestion, with Direct-zol RNA Kit (Zymo Research). Electrophoresis was conducted under the same non-denaturing conditions as described above and stained with GelStar (Lonza). ssRNA ladder (NEB N0362S) was used in all electrophoresis experiments.

### Particle-templated ClickSeq

Fifty micrograms of purified particles were used directly as a template in the reverse transcription reaction, without any RNA extraction. The ClickSeq library preparation generally follows previously developed methods (22, 29, 30), with several modifications to sequence exposed RNA in native particles. In brief, to preserve the particle integrity, a pre-RT denaturing step was removed (typically at 75°C for 10 min). Instead, purified particles were mixed with 5 pM of random hexamer primer with a partial i7 Illumina adapter and 1 µL of 10 mM AzNTPs:dNTPs mixture. A ratio of 1:5 AzNTPs:dNTPs was used to obtain a short distribution of cDNA fragments, which minimizes premature termination of reverse transcription due to possible RNA structure or protein interactions or potential elongation-induced stripping of RNA molecules from the remaining capsid shell. The RT reaction was conducted with SuperScript III reverse transcriptase (Invitrogen) with the manufacturer’s protocol and incubated at 50°C for 40 min. After reverse transcription, the products were treated with RNase H (37°C for 20 min), as per the standard ClickSeq protocol (30). The 3′-azido-terminated cDNAs were “Click”-ligated to a 5′-alkyne adapter, which also contained a 12N unique molecular identifier (UMI). The rest of the library preparation, including purification, final PCR amplification, and multiplexing, was detailed previously (29, 30).

Extracted and purified FHV RNAs were used with the same PT-ClickSeq method and modifications stated above to set a baseline control for read coverage of the fully exposed RNA template.

### Bioinformatics

For single-end sequencing data, the raw Illumina reads were first processed with *fastp* (61) to remove Illumina adapters, perform basic quality control, and assign unique molecular identifiers (UMIs) to reads (*-a AGATCGGAAGAGC -l 30 -U –umi_loc read1 –umi_len 14*). This is followed by mapping reads to the FHV genome with *bowtie2* (62) (*--local*). In order to control for PCR bias, *umi-tools* (63) was used (dedup --method=standard). Genome coverage information was analyzed with *samtools* (64) (*mpileup*).

For the paired-end sequencing data, *FASTX_toolkit* (http://hannonlab.cshl.edu/fastx_toolkit/) was used to apply an additional trimming step to the 5′ end of R2 reads, to remove the random hexamer plus one additional nucleotide as a buffer (*fastx_trimmer: -f 8*). The priming site was revealed from the R2 reads using BEDTools (65) (*genomecov -bga −5 -strand +*). Since R2 reads are complementary to R1 or the sense strand of the genome, this reports the 5′-most nucleotide of R2 reads after trimming (the 5′-8th nucleotide before trimming), which also corresponds to the 3′-most nucleotide on the genomic sense.

### Viral photo-activatable ribonucleoside crosslinking

Passage 1 (P1) FHV was used to infect naïve cells at an MOI = 1. As previously described (23), 100 µM of 4-thiouridine (Sigma-Aldrich, in DMSO) was supplied to infected cells at both 0 hpi and 16 hpi, to reach a final concentration of 200 µM. Infected cells and supernatant were harvested at 40 hpi. To fully release intracellular virions, the cell/supernatant was supplemented with 1% Triton X-100 and was allowed to go through one freeze-thaw cycle. 4SU-containing FHV particles were purified with the method stated above.

The purified 4SU-containing FHV (4SU+) was then heated to induce disassembly intermediates with the method described above. After heating and generation of disassembly intermediates, each type of particle was further purified with 100 K MWCO (PES) membrane filtration, which removes any free capsid/RNA fragments to prevent non-specific crosslinking. For each particle type (wt, eluted, puff), the 4SU-containing virus was separated into two pools: one pool underwent UV crosslinking (365 nm at 0.15 J/cm^2^), proteinase K digestion (8U, 37°C, 30 min) and RNA extraction (Zymo Direct-zol RNA kit) to yield the crosslinked particles (CL+); and the other pool only underwent proteinase K digestion and RNA extraction to yield the non-crosslinked controls (CL−).

The NGS library preparation of RNA samples extracted from CL+ and CL− particles is described previously (23). One important improvement is that this study utilized a 12N UMI adapter to be click-ligated with azido-terminated cDNA, which allows for de-duplication and bioinformatic control of PCR artifacts. Raw sequencing reads were processed the same way as single-end reads, as stated above. Pileup files were generated using *samtools*, and the nucleotide frequency and mismatch rate at each genomic position were analyzed with *vPARCL-call* (https://github.com/andrewrouth/vPARCL_call).

The vPAR-CL signal is defined as the fold change between crosslinked samples (CL+) and non-crosslinked controls (CL−). Biological triplicates of both CL+ and CL− were included for each particle type. In order to ensure the quality of vPAR-CL data, a minimum sequencing depth threshold was set to 1,000 de-duplicated reads.

## Data Availability

The raw FASTQ sequencing data of this study are available in the NCBI sequence read archive (SRA) with accession number: PRJNA1098227.

## SUPPLEMENTAL MATERIAL

Supplemental material jvi.00053-26-s0001.pdf Fig. S1 to S5 and Table S1.

Table S2 jvi.00053-26-s0002.xlsx PT-ClickSeq priming positions versus vPAR-CL signals.

ASM does not own the copyrights to Supplemental Material that may be linked to, or accessed through, an article. The authors have granted ASM a non-exclusive, world-wide license to publish the Supplemental Material files. Please contact the corresponding author directly for reuse.
