# Supplementary material for: Ordered release of genomic RNA during icosahedral virus disassembly
Source: J Virol. 2026 Apr 23;100(5):e00053-26. doi: 10.1128/jvi.00053-26 (PMC13185586; doi:10.1128/jvi.00053-26)
Supplement: Supplemental material — Fig. S1 to S5 and Table S1. [file jvi.00053-26-s0001.pdf]

Ordered release of genomic RNA during icosahedral virus disassembly
- Supplementary Figures and Table

Yiyang Zhou[1,2,4*], Andrew L. Routh[3,4,5,6,*]

Department of Pediatrics, Emory University, Atlanta, Georgia, USA

Department of Microbiology and Immunology, The University of Texas Medical Branch, Galveston, Texas, USA

Department of Immunology and Microbiology, Scripps Research, La Jolla, California, USA

Department of Biochemistry and Molecular Biology, The University of Texas Medical Branch, Galveston, Texas, USA

Sealy Center for Structural Biology and Molecular Biophysics, The University of Texas Medical Branch, Galveston, Texas, USA

Institute for Human Infections and Immunity, University of Texas Medical Branch, Galveston, Texas, USA

*To whom correspondence should be addressed.

Supplemental Figure S1

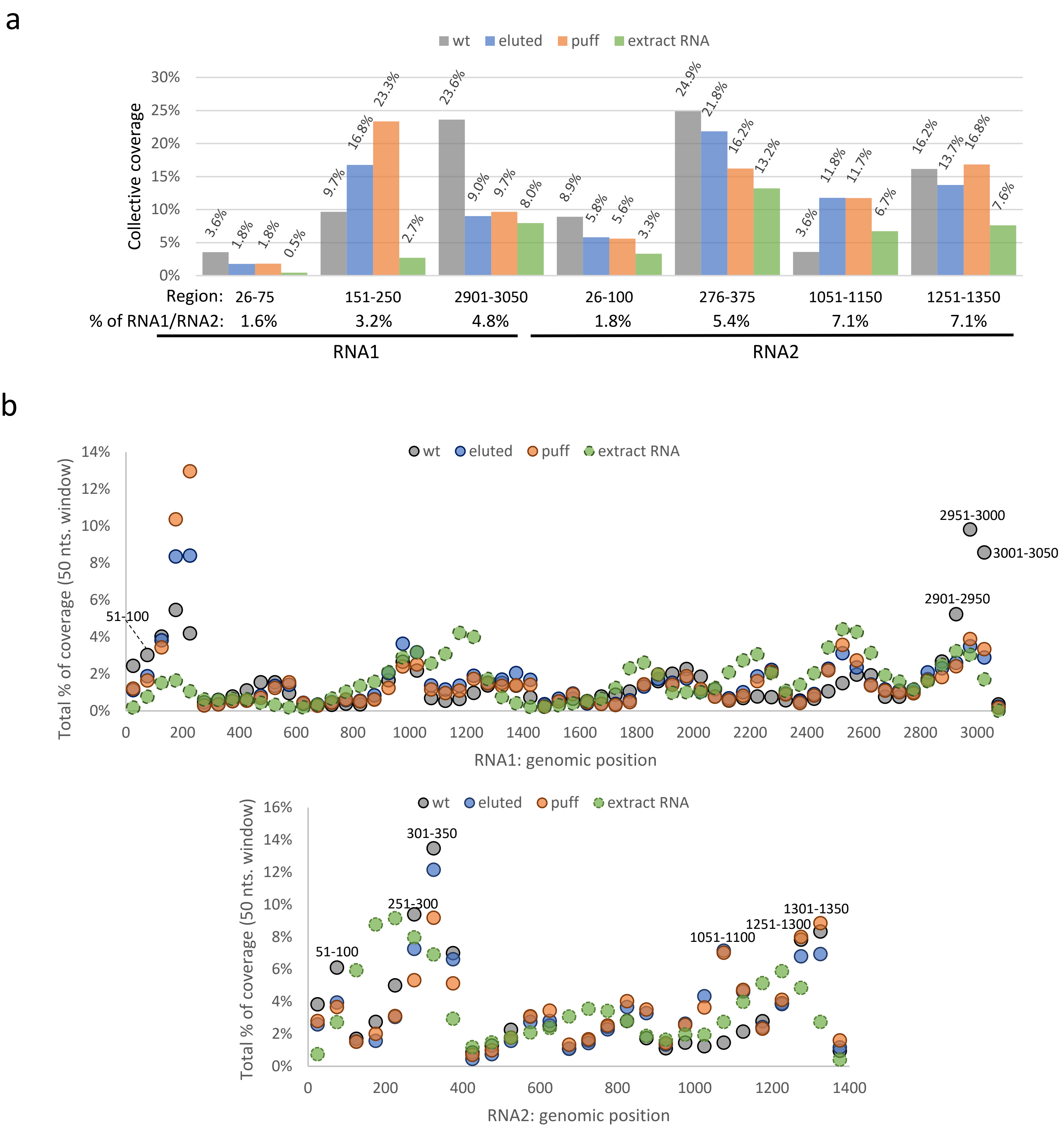

Supplemental Figure S1. Differential genome coverage of wt, eluted and puff particles. (**a**) The summed percentage coverage of specific genomic regions. (**b**) Each datapoint represents the summed percentage of coverage of a 50 nts window (e.g. nts 1-50).

Supplemental Table S1

	RNA1: 26-75 (1.6% of RNA1)				RNA1:151-250 (3.2% of RNA1)				RNA1:2901-3050(4.8% of RNA1)			
	wt	elute	puff	extr.RNA	wt	elute	puff	extr.RNA	wt	elute	puff	extr.RNA
collective coverage	3.56%	1.77%	1.81%	0.50%	9.66%	16.76%	23.33%	2.70%	23.63%	9.00%	9.66%	8.00%
95% CI	3.1%-4.02%	1.43%-2.11%	0.9%-2.71%		9.26%-10.07%	15.61%-17.92%	21.25%-25.41%		22.72%-24.54%	8.79%-9.2%	9.4%-9.92%	

	RNA1: 26-100 (1.8% of RNA2)				RNA2:276-375 (5.4% of RNA2)				RNA2:1051-1150(7.1% of RNA2)				RNA2:1251-1350 (7.1% of RNA2)			
	wt	elute	puff	extr.RNA	wt	elute	puff	extr.RNA	wt	elute	puff	extr.RNA	wt	elute	puff	extr.RNA
collective coverage	8.92%	5.80%	5.61%	3.30%	24.87%	21.84%	16.20%	13.20%	3.60%	11.78%	11.75%	6.70%	16.16%	13.73%	16.84%	7.60%
95% CI	8.39%-9.44%	5.19%-6.41%	3.67%-7.56%		23.85%-25.89%	21.36%-22.31%	15.83%-16.57%		3.46%-3.75%	11.42%-12.13%	10.64%-12.86%		15.82%-16.5%	13.47%-13.99%	16.71%-16.97%	

Supplemental Table S1. Differential genome coverage of wt, eluted and puff particles. The seven regions of RNA1 and RNA2 (from Supplemental Figure S1), their collective coverage in wt, elute, puff particles, and 95% confidence interval (CI).

Supplemental Figure S2

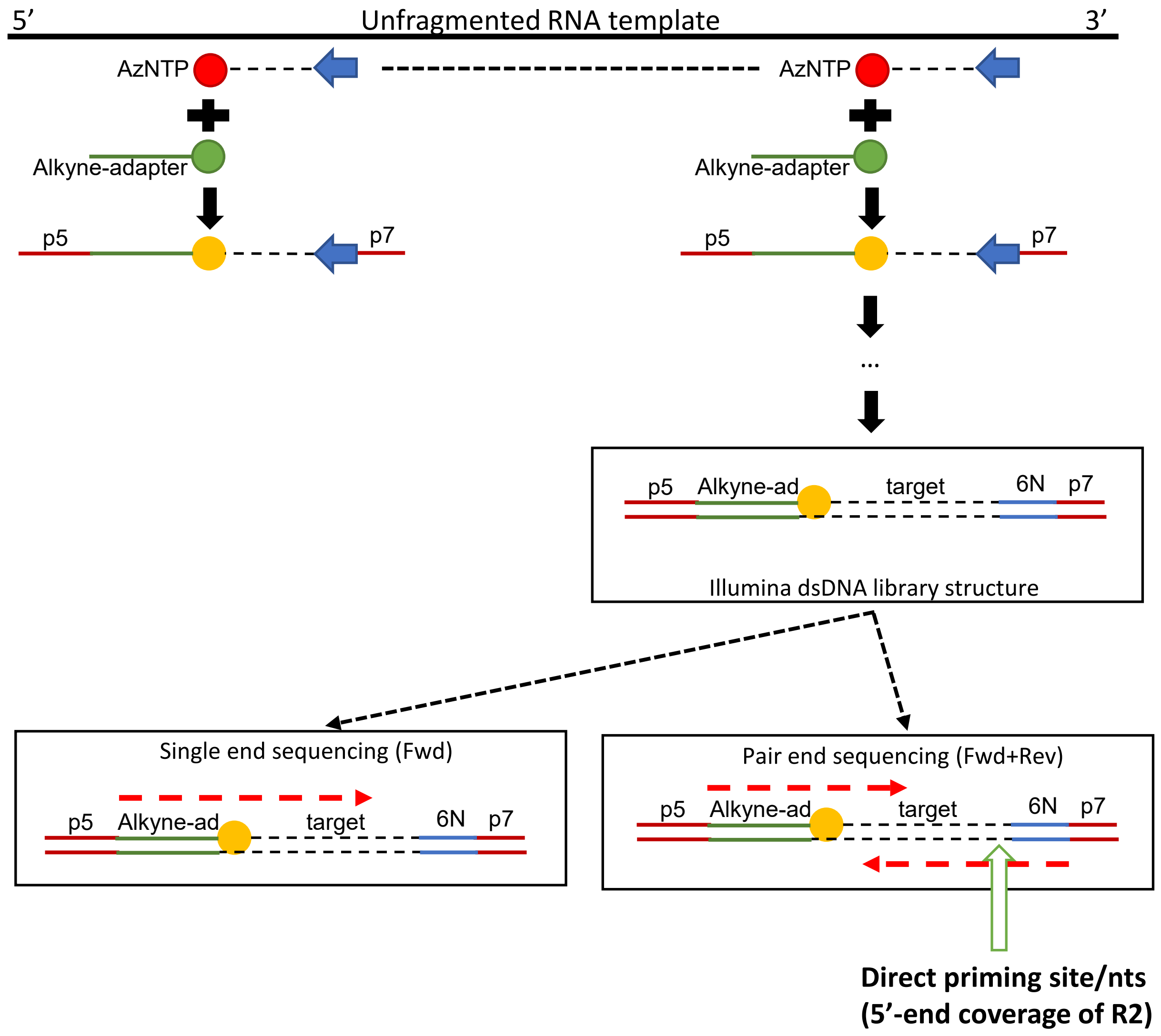

Supplemental Figure S2. Rationale of using paired-end sequencing to reveal priming sites. Single-end sequencing reveals information from the 5' end of the 'forward' read of stranded libraries (R1). The priming site in the reverse-transcription reaction is located at the 5' end of the 'reverse' read of stranded libraries (R2).

Supplemental Figure S3.

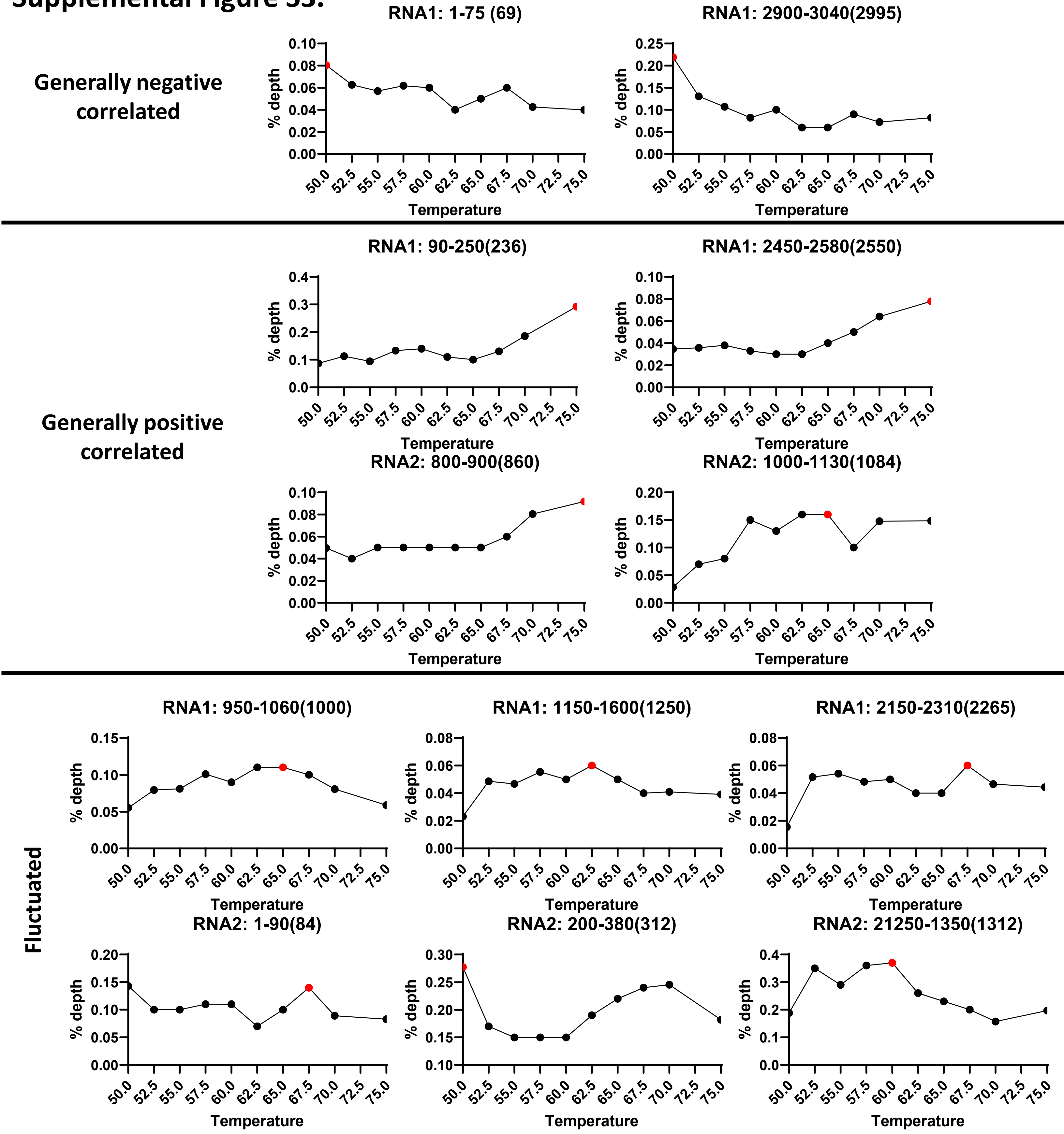

Supplemental Figure S3. Representative regions with different temperature sensitivity. Here shows the representative regions of **Figure 3**, their representative genomic positions (in parentheses) and general trends of coverage at the representative positions. Red dot: peak coverage.

Supplemental Figure S4

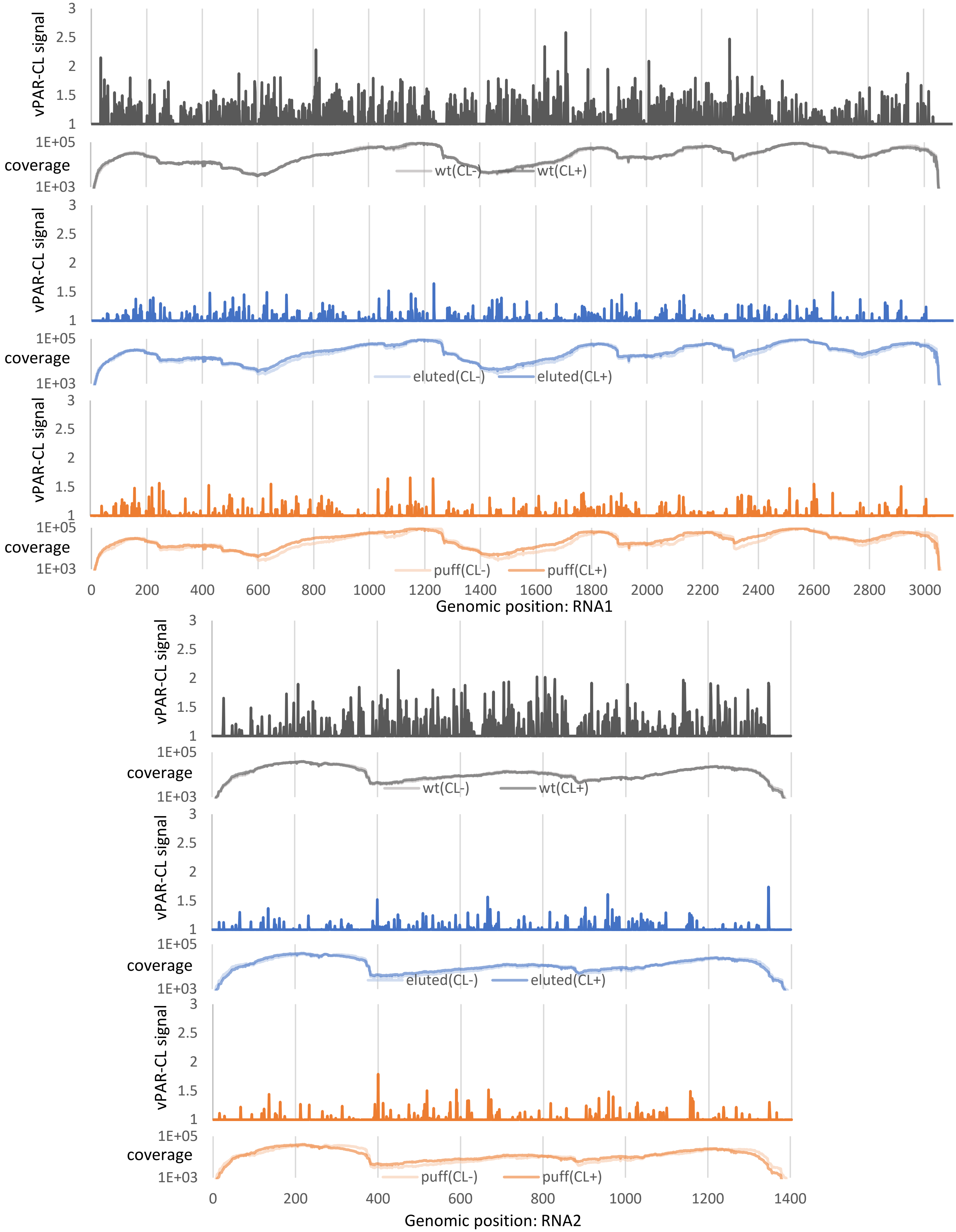

Supplemental Figure S4. Virus Photoactivatable Ribonucleoside Cross-Linking (vPAR-CL) signals of wt, eluted or puff particles. Signals were generated from the average of 4 independent experiments. The average coverage showed even sequencing depth and comparable read coverage between control groups (CL-) and crosslinked groups (CL+).

Supplemental Figure S5

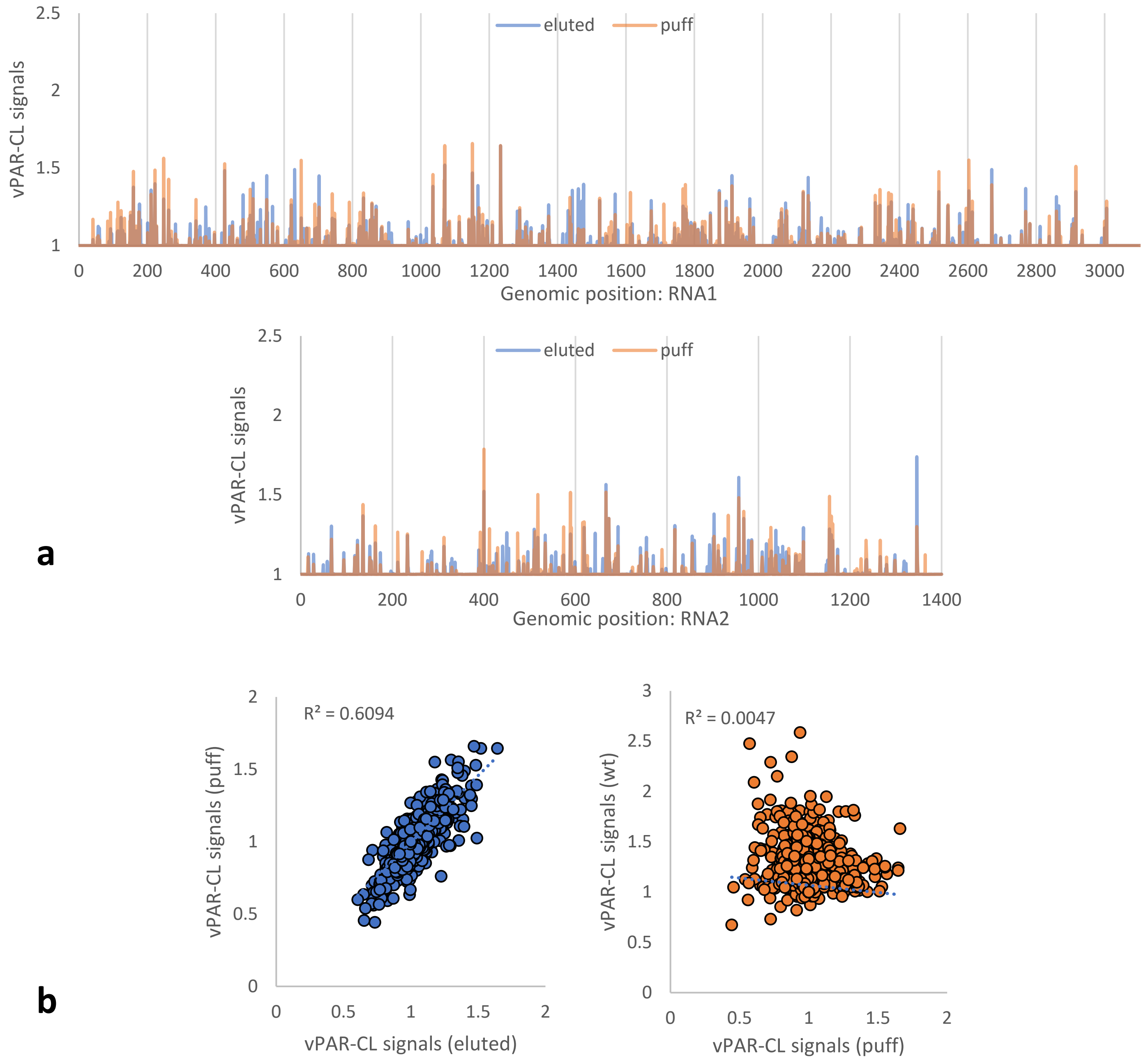

Supplemental Figure S5. Eluted and puff particles showed comparable RNA-capsid interaction patterns. (a) vPAR-CL signals from puff and eluted particles showed substantial overlap. (b) vPAR-CL signals of eluted and puff particles showed strong linear correlation (Pearson r=0.78). This is contrasted to the non-linear correlation between the vPAR-CL profiles of puff and wt particles (Pearson r = -0.06).
